# The Dynamic Roles of the Inner Membrane Complex in the Multiple Stages of the Malaria Parasite

**DOI:** 10.3389/fcimb.2020.611801

**Published:** 2021-01-08

**Authors:** Josie Liane Ferreira, Dorothee Heincke, Jan Stephan Wichers, Benjamin Liffner, Danny W. Wilson, Tim-Wolf Gilberger

**Affiliations:** ^1^ Centre for Structural Systems Biology, Hamburg, Germany; ^2^ Heinrich Pette Institut, Leibniz-Institut für Experimentelle Virologie, Hamburg, Germany; ^3^ Bernhard Nocht Institute for Tropical Medicine, Hamburg, Germany; ^4^ University of Hamburg, Hamburg, Germany; ^5^ Research Centre for Infectious Diseases, School of Biological Sciences, University of Adelaide, Adelaide, SA, Australia; ^6^ Burnet Institute, Melbourne, VIC, Australia

**Keywords:** malaria, *Plasmodium*, Apicomplexa, Alveolata, inner membrane complex, membrane dynamics

## Abstract

Apicomplexan parasites, such as human malaria parasites, have complex lifecycles encompassing multiple and diverse environmental niches. Invading, replicating, and escaping from different cell types, along with exploiting each intracellular niche, necessitate large and dynamic changes in parasite morphology and cellular architecture. The inner membrane complex (IMC) is a unique structural element that is intricately involved with these distinct morphological changes. The IMC is a double membrane organelle that forms *de novo* and is located beneath the plasma membrane of these single-celled organisms. In *Plasmodium* spp. parasites it has three major purposes: it confers stability and shape to the cell, functions as an important scaffolding compartment during the formation of daughter cells, and plays a major role in motility and invasion. Recent years have revealed greater insights into the architecture, protein composition and function of the IMC. Here, we discuss the multiple roles of the IMC in each parasite lifecycle stage as well as insights into its sub-compartmentalization, biogenesis, disassembly and regulation during stage conversion of *P. falciparum*.

## Introduction

The Apicomplexa represent a phylum of eukaryotic, single celled organisms that include human (i.e., *Plasmodium* spp., *Toxoplasma gondii*, and *Cryptosporidium* spp.) and livestock (i.e., *T. gondii*, *Eimeria* spp., and *Babesia* spp.) parasites with a severe impact on global health and socio-economic development. Human malaria parasites, *Plasmodium* spp., are the most medically important member of this distinct phylogenetic group and cause more than 400,000 deaths per year (WHO, 2018). Antimalarial resistant *P. falciparum*, the most lethal human malaria parasite species, are spreading (Dondorp et al., 2009; Imwong et al., 2017; Woodrow and White, 2017) and no efficacious vaccine has been developed to date. The devasting impact on endemic communities due to malaria has the potential to worsen with climate change and disruption of malaria control measures from outbreaks of other infectious diseases such as SARS-CoV-2 and Ebola (Rogerson et al., 2020; Sherrard-Smith et al., 2020).

**Figure 1:**
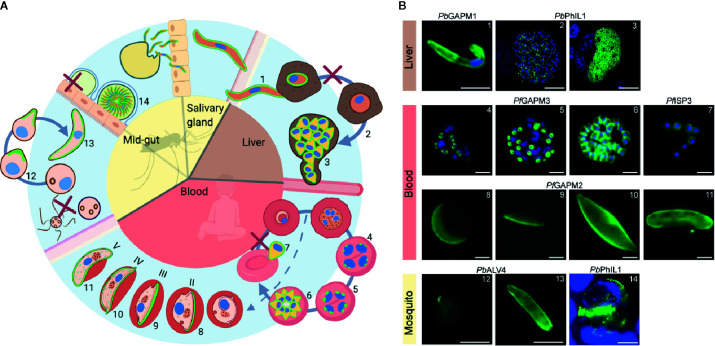
IMC dynamics through the malaria parasite life cycle. **(A)** Schematic representation of the lifecycle through the mosquito and human host. The IMC in different stages is highlighted in green. IMC disassembly is marked with a red cross. **(B)** Fluorescent microscopy images showing the IMC (green) at different stages in the parasite lifecycle. Specific IMC-GFP fusion proteins are indicated above the images. Blue; DAPI stain. Numbers in B refer to those in **(A)** 1 = Sporozoite (Kono et al., 2012), 2 = IMC biogenesis during liver cell schizogony (Saini et al., 2017), 3 = mature liver schizont (Saini et al., 2017), 4–6 = IMC biogenesis during schizogony in erythrocytes (Kono et al., 2012), 7 = free merozoites (Hu et al., 2010), 8–11 = developing IMC during gametocytogenesis (Kono et al., 2012), 12–13 = IMC biogenesis during ookinetogenesis (Kono et al., 2012), 14 = oocyst (Saini et al., 2017). Scale bars, 2 µm (micrographs 4–11) and 5 µm (micrographs 1, 12, 13, 14) 10 µm (micrographs 2, 3).

Human malaria infection begins after a bite of a female *Anopheles* spp. mosquito that injects the parasite into the skin (**Figure 1**). These parasites, called sporozoites, actively enter blood vessels and reach the liver where they invade hepatocytes. Within hepatocytes, the elongated sporozoites transform into spherical hepatic stages that replicate *via* multiple fission events and produce thousands of merozoites contained in host-cell derived vesicles known as merosomes (Stanway et al., 2011). The merozoites are released from the liver before they rapidly invade and multiply within red blood cells (RBCs); with this occurring repeatedly every ~48 h for *P. falciparum*. Mass proliferation in blood stages is responsible for the clinical manifestations of malaria. Some RBC-infecting parasites differentiate into sexual forms called gametocytes, which can be taken up during the blood meal of another mosquito. Male and female gametocytes fuse within the mosquito midgut and form a motile ookinete that transmigrates the mosquito midgut epithelium and differentiates into an oocyst. Oocyst maturation results in the formation of up to 2,000 sporozoites (Stone et al., 2013), which in turn are transmitted to the host during another blood meal of the mosquito.

In this review we walk through the malaria parasite’s lifecycle and focus on the role of the inner membrane complex (IMC) as the parasite transitions between various host cells such as RBCs, hepatocytes and mosquito midgut cells. The double membrane IMC organelle underlies the plasma membrane (PM) and is present in four of the morphologically distinct stages of the parasite’s lifecycle. These different parasite stages are not only characterized by their distinct physiology, but also by dramatic changes in size, shape and cellular architecture, facilitating growth and multiplication in diverse cellular environments. Here we primarily focus on the IMC of the human pathogen *P. falciparum*, with additional data from other *Plasmodium* spp. and *T. gondii* included to expand and sharpen our functional understanding of this unique membranous system.

## The Inner Membrane Complex: A Characteristic Cellular Structure Shared Across the Alveolata

*Plasmodium* spp., along with *T. gondii*, ciliates, and dinoflagellates are members of the Alveolata, a group of diverse unicellular eukaryotes. Evidence for a relationship between the diverse organisms that make up this phylogenetic supergroup only became clear from rRNA-sequence derived phylogenetic analysis (Wolters, 1991). Identification of a double membraned organelle below the PM (together called a pellicle) as a synapomorphic morphological feature, originating in ancestral Alveolata and shared across the supergroup, supports the evolutionary relationship between these unicellular eukaryotes (Cavalier-Smith, 1993).

The double membrane organelle underlying the PM, consisting of a series of flattened vesicles and interacting proteins, termed alveoli in ciliates, amphiesma in dinoflagellates and the IMC in the Apicomplexa, fulfills various functions depending on the organism’s diverse lifestyle and habitat. In aquatic ciliates and dinoflagellates, the alveoli play a structural role and can serve as a calcium storage organelle (Länge et al., 1995; Kissmehl et al., 1998; Ladenburger et al., 2009). In the endoparasites belonging to the Apicomplexa, the IMC has evolved additional functions that facilitate the unique strategies the parasites use to survive. As in the ciliates and dinoflagellates, the IMC of apicomplexan parasites provides structural support. However, the IMC also acts as a scaffold during the formation of daughter cells (Kono et al., 2012) and anchors the actin-myosin motor used for the gliding motility that powers host-cell invasion of these parasites, known as the glideosome (Keeley and Soldati, 2004). Like the secretory organelles (Rhoptries, Micronemes, and dense granules)—that form the core of the apical complex and have roles in motility/host cell invasion—the IMC is formed *de novo* during cytogenesis, starting at the apical pole of the parasite, and is most likely derived from Golgi vesicles (Bannister et al., 2000).

The large diversity in the functions and physiology of the pellicle are not limited to just the groups across the Alveolata, as the biology of the IMC also differs markedly between different apicomplexan parasites. Two examples of this can be found when comparing the IMC of the well-studied parasites *P. falciparum* and *T. gondii*. In the asexual cycle of *P. falciparum* malaria parasites, IMC biogenesis occurs *de novo* during schizogony (Kono et al., 2012). In *T. gondii*, the IMC is recycled from the mother cell into the forming daughter cells along with *de novo* biogenesis of new IMC double membrane during endodyogeny (Ouologuem and Roos, 2014). In *T. gondii*, the IMC and its associated glideosome are important in egress (Frénal et al., 2010) while this does not appear to be the case in *Plasmodium* spp. (Perrin et al., 2018).

The diversity in the IMC goes further with differences seen between parasites in different *Plasmodium* species (Ngotho et al., 2019) and beyond this to large morphological and functional differences in the IMC between each unique *P. falciparum* parasite stage as we discuss in this review.

## Defining The Inner Membrane Complex: Protein Composition and Complex Formation

To date ~45 IMC proteins have been identified in *Plasmodium* spp. (**Table 1**). Phylogenetic profiling of known IMC proteins has shown that the evolution of the IMC involved the repurposing of several ancestral eukaryotic proteins, such as coronin, together with the innovation of alveolate-specific proteins that occurred early in the Alveolata lineage such as the alveolins (Kono et al., 2012). More recent evolution of *Plasmodium* spp. specific proteins such as PF3D7_1345600 has led to a set of taxon-specific IMC proteins that facilitate the adaptation of the IMC to taxon-specific niches and functions. This evolutionary diversity mirrors the spectrum of functionalities provided by the IMC, from key roles in basic structural integrity for all Alveolates to the machinery that allows endoparasites to invade into and divide within specific host cells.

**Table 1:** *Plasmodium* spp. IMC proteins and other apicomplexan IMC proteins with *P. falciparum* homologs.

	Short ID	Gene ID	TM	Functional domains	References
	ALV1/IMC1a	PF3D7_0304000	No	Alveolin repeat	Khater et al., 2004 ^Pb^; Gould et al., 2008; Al-Khattaf et al., 2017 ^Pb^
	ALV2/IMC1e	PF3D7_0304100	No	Alveolin repeat	Kono et al., 2012; Tremp et al., 2014 ^Pb^
	ALV3/IMC1h	PF3D7_1221400	No	Alveolin repeat	Kono et al., 2012; Volkmann et al., 2012 ^Pb^; Coghlan et al., 2019 ^Pb^
	ALV4/IMC1g	PF3D7_0525800	No	Alveolin repeat	Hu et al., 2010; Kono et al., 2012; Gao et al., 2018 ^Py^
	ALV5/IMC1c	PF3D7_1003600	No	Alveolin repeat	Hu et al., 2010; Kono et al., 2012; Tremp et al., 2014; Tremp et al., 2017 ^Pb^
	ALV6/IMC1f	PF3D7_1351700	No	Alveolin repeat	Al-Khattaf et al 2015 ^Pb^; Gao et al., 2018 ^Py^
	ALV7/Pf77/IMC1j	PF3D7_0621400	No	Alveolin repeat	Kono et al., 2012; Gao et al., 2018 ^Py^
	IMC1b	PF3D7_1141900	No	Alveolin repeat	Tremp et al., 2008 ^Pb^
	IMC1d	PF3D7_0708600	No	Alveolin repeat	Al-Khattaf et al., 2015 ^Pb^
	IMC1i	PF3D7_0823500	No	Alveolin repeat	Kaneko et al., 2015 ^Pb^; Gao et al., 2018 ^Py^
	IMC1k	PF3D7_1341800	No	Alveolin repeat	Al-Khattaf et al., 2015 ^Pb^; Gao et al., 2018 ^Py^
	IMC1l	PF3D7_1417000	No	Alveolin repeat	Al-Khattaf et al., 2015 ^Pb^; Gao et al., 2018 ^Py^; Gao et al., 2018; Kumar et al., 2019 ^Py^
	IMC1m	PF3D7_1028900	Yes	Alveolin repeat	Al-Khattaf et al., 2015 ^Pb^; Gao et al., 2018 ^Py^
	ELC	PF3D7_1017500	No	EF Hand domain	Green et al., 2017
	GAP40	PF3D7_0515700	Yes (10)	None identified	Green et al., 2017
	GAP45	PF3D7_1222700	No	None identified	Baum et al., 2006
	GAP50	PF3D7_0918000	Yes (2)	Metallo-dependent phosphatase	Baum et al., 2006; Yeoman et al., 2011
	GAPM1	PF3D7_1323700	Yes (6)	None identified	Bullen et al., 2009; Hu et al., 2010; Kono et al., 2012
	GAPM2	PF3D7_0423500	Yes (6)	None identified	Bullen et al., 2009; Hu et al., 2010; Kono et al., 2012
	GAPM3	PF3D7_1406800	Yes (6)	None identified	Bullen et al., 2009; Kono et al., 2012
	MTIP	PF3D7_1246400	No	EF-hand domain	Bergman et al., 2003 ^Py^; Green et al., 2006; Dearnley et al., 2012
	MyoA	PF3D7_1342600	No	Myosin	Baum et al., 2006; Siden-Kiamos et al., 2011; Robert-Paganin et al., 2019; Blake et al., 2020 ^Pb^
	BCP1	PF3D7_1436200	No	None identified	Rudlaff et al., 2019
	BTP1	PF3D7_0611600	Yes (4)	None identified	Kono et al., 2016
	BTP2	PF3D7_0704100	Yes (7)	None identified	Rudlaff et al., 2019
	CINCH	PF3D7_0407800	No	None identified	Rudlaff et al., 2019
	HADO/HAD2	PF3D7_1205200	No	HAD phosphatase	Akinosoglou et al., 2015; Engelberg et al., 2016 ^Pb^
	Morn1	PF3D7_1031200	No	MORN repeats	Ferguson et al., 2008; Kono et al., 2016; Rudlaff et al., 2019
	ATRP	PF3D7_0410600	No	Armadillo repeats	Amlabu et al., 2020
	Coronin	PF3D7_1251200	No	WD40 repeats	Bane et al., 2016
	DHHC1	PF3D7_0303400	Yes (5)	DHHC Palmitoyltransferase, ankyrin repeats	Wetzel et al., 2015
	DHHC2	PF3D7_0609800	Yes (4)	DHHC Palmitoyltransferase, palmitoylated	Wang et al., 2020 ^Py^; Santos et al., 2015
	DHHC3	PF3D7_1121000	Yes (4)	DHHC Palmitoyltransferase	Frénal et al., 2013; Hopp et al., 2016 ^Pb^
	DHHC9	PF3D7_1115900	Yes (4)	DHHC Palmitoyltransferase	Tay et al., 2016
	G2	PF3D7_0929600	Yes (1)	EF hand domains, palmitoylated	Tremp et al., 2013 ^Pb^
	GAMER	PF3D7_0805200	No	None identified	Kaneko et al., 2015 ^Pb^
	ISP1	PF3D7_1011000	No	None identified	Wetzel et al., 2015; Poulin et al., 2013 ^Pb^; Gao et al., 2018 ^Py^
	ISP3	PF3D7_1460600	No	None identified	Hu et al., 2010; Poulin et al., 2013 ^Pb^; Gao et al., 2018 ^Py^; Kono et al., 2012; Wetzel et al., 2015
	NIPA	PF3D7_0522600	Yes (8)	Magnesium transporter	Hu et al., 2010; Kono et al., 2012
	NN	PF3D7_1345600	No	None identified	Hu et al., 2010; Kono et al., 2012
	NN	PF3D7_1445700	No	None identified	Birnbaum et al., 2017
	PhIL1	PF3D7_0109000	No	None identified	Kaneko et al., 2015; Schneider et al., 2017; Campelo Morillo et al., 2020 ^Pb^; Saini et al., 2017 ^Pb^
	PIP1	PF3D7_1355600	No	None identified	Schneider et al., 2017
	PIP2	PF3D7_1431100	No	None identified	Kono et al., 2012; Schneider et al., 2017
	PIP3	PF3D7_1430800	No	None Identified	Kono et al., 2012; Schneider et al., 2017
	SIP	PF3D7_0510300	No	None identified	Lentini et al., 2015 ^Tg^
	IMC20	PF3D7_1447500	No	None identified	Chen et al., 2015 ^Tg^
	IMC27	PF3D7_0518900	No	None identified	Chen et al., 2015 ^Tg^
	FBXO1	PF3D7_0619700	No	F-box domain	Baptista et al., 2019 ^Tg^
	AC5	PF3D7_0722900	No	None identified	Chen et al., 2015 ^Tg^
	ISC1	PF3D7_1341500	No	Palmitoylated	Chen et al., 2015 ^Tg^
	ISC3	PF3D7_1431900	Yes (8)	Choline transporter	Chen et al., 2015 ^Tg^
	MSC1b	PF3D7_1407700	No	None identified	Lorestani et al., 2012 ^Tg^
	PP7/AC6	PF3D7_1423300	No	Serine/Theronine phosphatase, EF hand domains	Chen et al., 2015 ^Tg^

A list of the currently identified IMC proteins in Plasmodium spp., along with the P. falciparum homologs of proteins that have been localized to the IMC in other apicomplexans. Proteins are grouped by location (blue, Alveolins; red, Glideosome; green, Basal complex; yellow, IMC) and ordered within groups by their name to emphasize proteins with potentially related functions/localization. In gray are Plasmodium falciparum homologs of IMC proteins identified in Toxoplasma gondii (Tg) but which have yet to be confirmed as IMC localized in Plasmodium spp. Transmembrane domain and functional domain data is based on predictions from PlasmoDB (Aurrecoechea et al., 2009). All references where work was not carried out in P. falciparum are noted (Pb, Plasmodium berghei; Py, Plasmodium yoelii). NN, not named. Additional information including the results of random mutagenesis of IMC proteins covered in this table are available at the online database www.plasmoddb.org.

The best studied group of IMC proteins are components of the motor complex that drives the locomotion of all motile parasite stages—also referred to as the “glideosome” (**Table 1**) (Webb et al., 1996; Opitz and Soldati, 2002; Baum et al., 2006). This actin-myosin motor complex powers the motility needed for transmigration, gliding, invasion and potentially egress (Frénal et al., 2010); the physiological trademark of the motile merozoite, sporozoite and ookinete stages of parasite development. Although multiple components of the glideosome such as the glideosome-associated proteins GAP40 GAP45, GAP50, GAP70, and GAPMs (Gaskins et al., 2004; Bullen et al., 2009; Frénal et al., 2010; Yeoman et al., 2011) together with Myosin A (MyoA) (Pinder et al., 1998; Baum et al., 2006; Ridzuan et al., 2012), the myosin A tail-interacting protein [MTIP in *Plasmodium* spp., or myosin light chain (MLC1) in *T. gondii*] (Herm-Götz et al., 2002; Bergman et al., 2003) and the “essential light chain 1” protein (ELC1) (Green et al., 2017) have been identified, a comprehensive and systemic identification of all components of the glideosome has not been performed. Recent years have uncovered some structural insights into this protein complex, but this improved understanding is still limited to individual components (Bosch et al., 2006; Bosch et al., 2007; Bosch et al., 2012; Boucher and Bosch, 2014) or to sub-complexes such as the trimeric structure composed of MyoA, ELC1 and MLC1 (Moussaoui et al., 2020; Pazicky et al., 2020) with no structural information on the entire glideosome complex.

Another interesting group of proteins are the so called alveolins, an Alveolata specific and conserved multi-protein family encompassing at least 13 proteins (**Table 1**) (Gould et al., 2008; Kono et al., 2012; Volkmann et al., 2012; El-Haddad et al., 2013; Tremp et al., 2014; Al-Khattaf et al., 2015). A representative alveolin was initially identified in *T. gondii* and named Inner Membrane Complex Protein 1 (TgIMC1) (Mann and Beckers, 2001). Alveolins are peripheral membrane proteins that constitute a subpellicular network of proteins located at the cytoplasmic face of the IMC. They are characterized by the presence of one or more highly conserved domains composed of tandem repeat sequences and were recognized as a unique protein family shared across all alveolates (Gould et al., 2008; Kono et al., 2012; El-Haddad et al., 2013; Al-Khattaf et al., 2015). Members of this family show stage specific expression patterns in *P. falciparum* and have been implicated in parasite morphogenesis and gliding motility at least in sporozoites and ookinetes, although the precise molecular details for these roles have yet to be elucidated for alveolins of *Plasmodium* spp. (Khater et al., 2004; Tremp et al., 2008; Volkmann et al., 2012). Stage specific transcriptomics in *P. falciparum* (López-Barragán et al., 2011; Gómez-Díaz et al., 2017; Zanghì et al., 2018) show that some alveolins are expressed across all lifecycle stages (e.g., IMC1a/ALV1, IMC1c/ALV5) while others seem to be absent in specific stages (e.g., IMC1f/ALV6 in gametocytes, IMC1l in asexual blood stages) or are exclusively expressed in one specific stage (e.g., IMC1i in ookinetes). A comprehensive detailed functional mapping of this multi-gene family across different stages of the *P. falciparum* lifecycle will be instrumental to understand the precise function of individual alveolins.

Beside the alveolins, other additional IMC associated peripheral membrane proteins like the IMC sub-compartment-proteins (ISPs) (Beck et al., 2010; Hu et al., 2010) have been characterized (Tonkin et al., 2004; Fung et al., 2012; Poulin et al., 2013; Gao et al., 2018; Wang et al., 2020). In contrast to integral membrane proteins of the IMC that are most likely trafficked *via* the ER and Golgi to their final destination (Yeoman et al., 2011), the majority of these peripheral membrane proteins are linked to the IMC membrane by their lipid anchors. For example, the ISP proteins as well as GAP45 depend on their N-terminal palmitoylation and myristoylation motif for membrane association (Wetzel et al., 2015; Wang et al., 2020). This occurs *via* co-translational and post-translation modification by the cytosolic N-myristoyltransferase (Gunaratne et al., 2000) and IMC embedded palmitoyl acyl transferases (Beck et al., 2013; Frénal et al., 2013; Wetzel et al., 2015). The alveolins, however, do not display a dual acylation motif and how they are trafficked to and interact with the IMC has yet to be described.

## Inner Membrane Complex Structure and Function Throughout the Plasmodium Life Cycle

### The Asexual Blood Stage

Each individual *P. falciparum* merozoite within the schizont is surrounded by the double-membrane IMC, which sits approximately 20 nm below the PM (Morrissette and Sibley, 2002). The IMC is described as a mono-vesicle in this stage and appears to be continuous in fluorescence microscopy images, although gaps or discontinuities in the merozoite IMC have been observed in cryo-preserved TEM images (Hanssen et al., 2013; Riglar et al., 2013). At each pole of the merozoite is a defined sub compartment of the IMC. At the apical end of the merozoite, the IMC is supported by subpellicular microtubules (SPMs, 2-3 per merozoite) which are anchored in and organized by a series of rings termed polar rings (Morrissette and Sibley, 2002). At the basal end of the merozoite an additional structure, referred to as the basal complex is located which was first described in *T. gondii* (Ferguson et al., 2008; Hu, 2008; Lorestani et al., 2010).

The *de novo* biogenesis of the IMC has been studied in some detail in *P. falciparum* by fluorescence microscopy (Yeoman et al., 2011; Dearnley et al., 2012; Kono et al., 2012; Ridzuan et al., 2012). This process occurs during schizogony where a new IMC is formed for each of the 16–32 individual daughter merozoites in a schizont (**Figure 1**). The IMC of the growing daughter cells is established at the apical end of each new merozoite prior to nuclear division. The nucleation of the nascent IMC is closely associated with the centrosome placing it in the center of the developing IMC structure (Kono et al., 2012). The budding IMC grows from a point at the apical end into a ring until, at the end of schizogony; it has expanded to completely cover the newly formed daughter cell. During this process, the basal complex marks the leading edge of the growing IMC and migrates from the apical pole of the daughter cell to the basal pole where it resides after the completion of schizogony (Kono et al., 2016). PfMORN1 (Ferguson et al., 2008; Kono et al., 2016), PfBTP1 (Kono et al., 2016), PfBTP2 (Rudlaff et al., 2019), PfBCP1 (Rudlaff et al., 2019), PfHAD2a (Engelberg et al., 2016), and PfCINCH (Rudlaff et al., 2019) are well established basal complex markers. Parasites deficient in PfCINCH have impaired segmentation of daughter cells, suggesting this protein has an essential role in contraction of the basal complex and pinching off of newly developed daughter cells (Rudlaff et al., 2019) and highlighting the important role of the IMC during cytokinesis. While in *P. falciparum*, how this contraction works remains unknown, in *T. gondii*, the apparent role of the myosin, MyoJ, in the contraction implies a mechanism involving the actin-myosin motor (Frénal et al., 2017).

Another process that remains mechanistically completely unknown is the rapid disassembly of the IMC after successful re-invasion of the merozoite into a new RBC. Once invasion is complete, the IMC as a central structure for cytokinesis and motility has outlived its purpose and needs to be disassembled to allow for growth and division of the parasite. The disassembly of the entire IMC may happen in as little as 15 min (Riglar et al., 2013) but definitely appears to be completed within 1 h. How this task is achieved, how it is regulated and what happens to the lipids and proteins from the now superfluous double-membrane structure remains to be explored.

### The Sexual Blood Stage: Gametocytes

To achieve transmission from the vertebrate host to the mosquito the parasite produces male and female gametocytes. Sexual stage conversion in *P. falciparum* is characterized by drastic morphological changes of the parasite that occur over approximately 10–12 days and includes five (I to V) morphologically distinct stages (**Figure 1**) (Fivelman et al., 2007). Fully mature gametocytes display a falciform shape, from which *P. falciparum* derives its name. During their maturation, gametocytes lengthen significantly with fully mature gametocytes being 8–12 µm in length, approximately six times bigger than merozoites. Early in gametocytogenesis, their distinct morphology permits the gametocyte to sequester and develop within the bone marrow. Once fully mature, gametocytes migrate back into circulation where their morphology enables them to avoid immune clearance during passage through splenic sinuses unlike asexual parasites (Joice et al., 2014; Lee et al., 2018; De Niz et al., 2018; Obaldia et al., 2018).

The double membrane IMC below the PM in gametocytes was first identified 40 years ago (Smalley and Sinden, 1977). In *P. falciparum* it is organized in distinct vesicles or plates numbering between 11 (Meszoely et al., 1987) and 13 (Kono et al., 2012; Schneider et al., 2017) per gametocyte. The IMC plates are thought to be connected *via* proteinaceous “sutures”, although no direct correlation between the distinct delineating lines seen in fluorescence microscopy and structures in electron microscopy have been made to date. The sutures are hypothesized to support the alignment of microtubules (Kaidoh et al., 1993; Dearnley et al., 2012; Kono et al., 2012) or to connect the IMC with the PM (Meszoely et al., 1987). Presently, two proteins (Pf3D7_1345600 and DHHC1) have been localized to the sutures in gametocytes (Kono et al., 2012; Wetzel et al., 2015), although no functional data connecting these proteins to their distinct localization is available.

Maturation of *P. falciparum* gametocytes can be accurately assessed by IMC formation. Stage I gametocytes are only distinguishable from asexual trophozoites on the molecular level, through expression of specific marker proteins such as Pfs16 (Bruce et al., 1994) with no IMC proteins detectable at this stage (Dearnley et al., 2012; Kono et al., 2012). Stage II gametocytes are characterized by elongation of the transversal microtubules and the start of IMC biogenesis (Sinden, 1981). IMC marker proteins such as PhIL1 (Schneider et al., 2017), GAP45 (Dearnley et al., 2012) and GAPM2, ISP3 and PF3D7_1345600 (Kono et al., 2012) appear as a spine-like structure with longitudinal orientation in close association with an array of microtubules. Three-dimensional SIM immunofluorescence microscopy shows a ribbon-like arrangement of PhIL1 around the microtubules (Schneider et al., 2017). Although still unclear, it is likely that microtubules provide a scaffold for the placement of the Golgi-derived IMC-bound vesicles that fuse to form the IMC. During stage III, the IMC membranes further wrap around the parasite and show thickened areas at the growing edge (Schneider et al., 2017). This then proceeds to stage IV where the parasite is maximally elongated and has a “banana-like” structure with the IMC completely surrounding the parasites. The final stage V is characterized by disassembly of the microtubule network until only small patches are observed below the IMC, a process initiated by an unknown signal. In parallel with this process, the parasite rounds up and the sutures become less pronounced (Dearnley et al., 2012; Schneider et al., 2017). Disassembly of the microtubule network leads to changes in the rigidity and deformation of the parasite (Dearnley et al., 2012) that promotes the reentering of these parasites from the bone marrow into the peripheral blood circulation and their uptake by the mosquito.

During uptake by the mosquito, the gametocytes are activated by external stimuli (reduced temperature, xanthurenic acid and pH rise), round up, egress from the RBC and transform into female macrogametes and exflagellated male microgametes. This transformation happens very rapidly (∼2 min) (Sologub et al., 2011) and is highly dependent on disassembly of the IMC as a prerequisite for rounding up. Again it is unclear how this process is initiated, what its molecular basis is, or the fate of the membrane following disassembly. An interesting observation is the formation of nanotubules between forming gametes after gametocyte activation (Rupp et al., 2011). These are membranous cell-to-cell connections that may facilitate mating of the male and female gametes. The authors provide an intriguing hypothesis, which proposes that these may be formed out of the recycled IMC-vesicles as their appearance is coincident with the disappearance of the IMC (Rupp et al., 2011). Interestingly, a recent study in *P. berghei* suggests that active trans-endothelial migration of gametocytes (De Niz et al., 2018) exhibit an as yet uncharacterized mode of actin-dependent deformability and/or motility that could also be dependent on the IMC.

Taken together, in contrast to its central role in motility in invasion-competent merozoites, ookinetes and sporozoites, the IMCs most prominent role during gametocytogenesis appears to be a structural one which drives this stage’s significant changes in size and shape (Dixon et al., 2012; Kono et al., 2012; Schneider et al., 2017).

### In the Mosquito: From Zygote to Ookinete

Following fusion of the activated male and female gametes in the midgut of mosquitoes, a diploid, non-motile zygote is formed which undergoes meiosis and transforms into immature ookinetes known as retorts (reviewed in Bennink et al., 2016). These intermediate cells develop and 20 h post gametocyte fusion are fully transformed into motile ookinetes (Siciliano et al., 2020), which transmigrate through the epithelial cells to settle beneath the basal lamina of the mosquito midgut (**Figure 1**).

Like in merozoite maturation in the asexual blood stage, the IMC is formed again *de novo* during ookinetogenesis, starting as a patch at the apical end of the zygote (**Figure 1**) (Tremp et al., 2008). The round zygotes undergo huge and rapid morphological changes, gaining a protrusion, elongating and maturing to finally form an elongated, crescent-shaped ookinete (Guttery et al., 2015; Bennink et al., 2016). The ookinete’s apical rings are formed at the same time and organize the SPM of > 40 microtubules (Bertiaux et al., 2020) under the IMC. These are initially seen at the apical protrusion of the zygote but extend during zygote elongation into a “dome-like” structure under the IMC (Wang et al., 2020). The dual acetylated peripheral IMC proteins ISP1 and 3 appear to serve here as a tether linking the SPM with the IMC in order to maintain a proper pellicle cytoskeleton. Congruently, ΔISP1 and ΔISP3 parasites show a reduced rate of zygote to ookinete differentiation, indicating that the IMC has an important role in transitioning from a round zygote to a crescent shaped ookinete (Wang et al., 2020).

Together with its role in mediating some of the large morphological changes that occur during the transformation from round, fertilized zygote to an elongated ookinete, another obvious role of the IMC is the facilitation of ookinete motility. Again, the IMC acts to anchor the glideosome, providing a stable structure from which to produce and exert forward motion. Motility is important for crossing the midgut and establishment of the replicative oocyst stage in the mosquito. Knock out of the IMC protein IMC1b/ALV5 led to reduced ookinete motility and therefore fewer oocysts produced in the mosquito (Tremp et al., 2008). After crossing the mosquito midgut, the ookinete differentiates into an oocyst. The transition from ookinete to early oocyst is also associated with loss of the IMC (Harding and Meissner, 2014) prior to sporogony. No details of this process are known yet, including what triggers and mediates disassembly and what happens to the large amount of the lipid and protein.

Relatively little is known about the architecture and protein composition of the IMC in these mosquito infecting stages. However, freeze fracture SEM images of ookinetes from the poultry parasite *P. gallinaceum* provide a glimpse of what appears to be a largely distinct IMC morphology from other stages (Alavi et al., 2003). The micrographs show a single vesicle punctured by multiple large pores. The pores have an external diameter of 43 nm and 12-fold symmetry. The single IMC vesicle appears to have a zig-zagged row of proteins connecting it along one edge, which has been termed a suture, as in gametocytes. What these pores are composed of, their function and why they have not been observed in other stages is currently unclear.

### Out of the Mosquito: Sporozoite Formation and Motility

Once in the basal lamina the ookinete develops into an oocyst (**Figure 1**). Sporogony leads to thousands of sporozoites forming within the ~14 days of oocyst development. During sporogony, again, the IMC is formed *de novo* around each new sporozoite. It appears to be a complex process as the parasite grows larger, transforms from a “solid phase” to a vacuolated phase, undergoes multiple nuclear divisions and forms multiple segregated sporoblasts (Terzakis et al., 1967). Each sporoblast within the oocyst acts as a center for the synchronous budding of hundreds of new sporozoites (Sinden and Matuschewski, 2005). At the initiation of budding, the microtubule organizing center is positioned at the cortex. In a process similar to IMC formation in the merozoite, the apical rings and microtubules are in place and the IMC grows toward the basal end of each elongating parasite until a final contraction at the basal end completes sporogony (Schrevel et al., 2008). The versatile sporozoite emerges from the oocyst (Frischknecht and Matuschewski, 2017), and makes its way to the mosquito salivary glands where it invades first the basal lamina and then the acinar cells (Stering and Aikawa, 1973).

The elongated sporozoite (10–15 µm) is highly motile and moves at speeds of over 2 µm/s (Münter et al., 2009) for multiple minutes. Motility remains after injection into the dermis by the mosquito (Vanderberg, 1974; Amino et al., 2006) until the parasites invade a blood vessel, travel to the liver and finally settle in a hepatocyte. The distinctive crescent sporozoite shape may be due to a linkage of myosin to the subpellicular network (SPN) below the IMC (Khater et al., 2004; Kudryashev et al., 2012)*. *In turn, this shape is likely to contribute to the distinct spiral gliding trajectories of the sporozoite (Akaki and Dvorak, 2005). This unusual gliding motility (Frischknecht and Matuschewski, 2017) depends on proteins of the TRAP (thrombspondin related anonymous protein) family (Sultan et al., 1997). TRAP proteins span the PM and possess a cytoplasmic tail domain that is believed to interact with actin, since mutations in this region led to defects in active locomotion (Ejigiri et al., 2012). TRAP proteins are released from micronemes to the sporozoite surface (Gantt et al., 2000; Carey et al., 2014) and serve as an adaptor and force transmitter between the target cells and the sporozoite.

The sporozoite stage has been studied by cryo-electron tomography, which allows a view of the IMC without artefacts from staining or slicing (Kudryashev et al., 2010). In these cells, the IMC sits approximately 30 nm below the PM and appears to be a highly homogenous flattened vesicle with minor discontinuities noted (Kudryashev et al., 2012).

### Into the Blood: Hepatic Schizogony and Merozoite Formation

After invasion of the hepatocyte, the sporozoite dedifferentiates from the elongated motile cell into a rounded cell, starting at its center. This rounding is accompanied by the disassembly of the IMC as well as the sporozoite’s subpellicular network (Jayabalasingham et al., 2010). Intriguingly, the rounding of the sporozoite and disassembly of the IMC does not require a host cell as it can also be triggered outside the cell at 37°C in serum (Kaiser et al., 2003). Early EM images of *P. berghei* in hepatocytes show the shrinkage of the IMC to cover half the cell at 24 hpi, with just a small section covered at 28 hpi (Meis et al., 1985). This view of IMC disassembly after sporozoite invasion of hepatocytes is supported by fluorescence microscopy studies showing the shrinkage of the IMC to one side of the cell (Kaiser et al., 2003). More recently, EM images appear to show the dispersal of a membrane thought to be the IMC into the parasitophorous vacuole (PV) in hepatocytes infected with *P. berghei*, which the authors suggest could be a rapid mechanism for clearing this organelle and allowing for the fast growth and multiplication of the parasite (Jayabalasingham et al., 2010). Importantly, IMC disassembly is a prerequisite for the first mass-proliferative stage of schizogony within the host (Meis et al., 1985; Stanway et al., 2011) resulting in up to 29,000 exoerythrocytic merozoites in *P. berghei* (Baer et al., 2007) and up to 90,000 in *P. falciparum* (Vaughan et al., 2012). How exactly the disassembly or expulsion of the IMC occurs and what controls it remains to be determined.

## Outlook

The IMC is a unique structural compartment that undergoes continuous and profound reconfiguration during the parasite’s lifecycle, mirroring the changes in stage specific architecture and motility that are reliant on IMC function. Although our understanding of the IMC has continuously increased over the years driven by molecular studies in *Plasmodium* spp. *as well as in T. gondii*, there are still large gaps in our knowledge and many unanswered questions in the field.

Many evolutionary questions still remain unanswered such as what selection pressures may have led to the evolution of the IMC in the ancestor of alveolates and what contribution did the IMC play in speciation. Clearly, the IMC was repurposed into a useful scaffold from which to drive cell invasion of apicomplexans. How this complex motor came to interact with the IMC scaffold may become apparent as we learn more about the early apicomplexan parasites and their move toward intracellular parasitism.

Many functional questions about the IMC in different stages of parasite development remain open. These include how IMC architecture at molecular resolution differs between stages, what the stage specificity of proteomes is and whether sub-compartmentalization and regulation of IMC assembly and disassembly is different in each stage. Another open question is how the different subcompartments of the IMC switch functions depending on their requirements. While some subcompartments and protein complexes, such as the basal complex and glideosome, have been partially characterized across different stages of the life cycle, such detailed studies have not yet been undertaken for others. Additional intriguing structures such as the apical annuli have been described in *T. gondii* that appear to be embedded in the IMC (Hu et al., 2006; Beck et al., 2010; Engelberg et al., 2020). Although there is currently no experimental evidence for the presence of such a structure in *Plasmodium* spp., a homolog of an apical annuli protein has been identified in the genome of *P. falciparum* that might serve as an “IMC pore” (Engelberg et al., 2020).

Another missing link is between the formation of the IMC and nuclear division. During mitosis, there appears to be a switch from local control of asynchronous rounds of nuclear division to global control. This final, global round of nuclear division occurs in parallel to IMC formation as well as with other processes of daughter cell formation. The molecular drivers of this process remain to be determined. In *T. gondii*, the bipartite centrosome acts as a signalling hub and coordinates the separation of nuclear division and daughter cell formation (Suvorova et al., 2015). However, although it can be speculated that a similar mechanism is likely to exist in *P. falciparum*, it has yet to be demonstrated.

A key question for future study is how the IMC’s large quantity of lipid and protein gets recycled or remade in each stage of the lifecycle. Other than a few hints from electron micrographs showing pieces of membranes below the PM in young rings (Riglar et al., 2013) or the apparent expulsion of membrane in hepatocytes (Jayabalasingham et al., 2010), neither of which have been confirmed to be of IMC origin, we have no clear understanding of what happens to the IMC after it is no longer needed at different stages of *Plasmodium* spp. development. A few different hypotheses could be imagined, including the IMC merging with the PM after selective proteolysis of IMC proteins by the ubiquitin-proteasome system, bulk degradation by macroautophagy or bulk expulsion of the whole organelle. Degradation followed by *de novo* biogenesis in each stage is energy intensive, making it tempting to imagine a mechanism where the lipids and proteins are recycled. However, whether and how the IMC double membrane and protein is recycled or synthesized across different stages of malaria parasite development, often within a very short time-frame (minutes), is another mystery of this versatile organelle that remains to be elucidated.

Finally, with recent advances in techniques, particularly of imaging methods, we expect that answering the questions outlined above is already or will soon become feasible. As the IMC lies so closely below the PM (~20–30 nm) and its two membranes are extremely close together (~10 nm), few tools have high enough resolution to distinguish the three membranes. Although data from FIB-SEM of plastic embedded samples has not yet reached the resolution required to clearly resolve the IMC in published data, this technique has huge potential for providing 3D overviews of cell morphology (Rudlaff et al., 2020). To obtain more detailed information and easily resolve the three membranes, cryo electron tomography of FIB-milled samples will be necessary. Although EM techniques provide the spatial resolution needed, they only give a snapshot in time. The processes that we have described here are dynamic and vary in their speed. They therefore also need to be studied with tools which provide superior spatial and temporal resolution over, e.g., the period of IMC assembly, motile function and disassembly. The implementation of novel imaging techniques into the malaria field, such as lattice light sheet microscopy, is promising and has the potential to provide high-resolution, temporal insights into the dynamic IMC.

## Author Contributions

All authors wrote and edited the manuscript. All authors contributed to the article and approved the submitted version.

## Funding

This work was partially supported by the Human Frontier Science Program LT000024/2020-L (JF), Hospital Research Foundation Fellowship (DW), Australian Government Research Training Program Scholarship (BL), DFG BA5213/3-1 (JW), DAAD/Universities Australia joint research co-operation scheme (T-WG, DW, BL, and DH), and a CSSB Seed Grant (KIF-2019/002).

## Conflict of Interest

The authors declare that the research was conducted in the absence of any commercial or financial relationships that could be construed as a potential conflict of interest.

The reviewer RT declared providing some of the pictures used in this review article, and confirms the absence of any other collaboration with the authors to the handling editor.
